# Health Literacy and Health Behavior: Associated Factors in Surabaya High School Students, Indonesia

**DOI:** 10.3390/ijerph18158111

**Published:** 2021-07-30

**Authors:** Junaidi Budi Prihanto, Faridha Nurhayati, Endang Sri Wahjuni, Ryota Matsuyama, Miwako Tsunematsu, Masayuki Kakehashi

**Affiliations:** 1 Department of Health Informatics, Graduate School of Biomedical and Health Science, Hiroshima University, Hiroshima 734-8553, Japan; rmatsuyama@hiroshima-u.ac.jp (R.M.); tsunematsu@hiroshima-u.ac.jp (M.T.); kakehashi@hiroshima-u.ac.jp (M.K.); 2 Department of Sport Education, Faculty of Sport Science, State University of Surabaya, Surabaya 60213, Indonesia; faridhanurhayati@unesa.ac.id (F.N.); endangwahjuni@unesa.ac.id (E.S.W.)

**Keywords:** health behavior, health literacy, health-promoting school, high school student, adolescent

## Abstract

(1) Background: The health behavior (HB) of adolescents develops in the school or family setting and plays an important role in their future health status. Health literacy (HL) has been identified as an important factor in modifying health behavior in addition to socioeconomic factors. health-promoting school (HPS) programs also have a significant role in providing students with the means of learning the importance of knowledge, behavior, and skills for a healthy lifestyle. (2) Method: This study aims to identify the association between HB, HL measured in comprehensive health literacy (CHL) and functional health literacy (FHL), HPS programs, and socioeconomic factors among high school students in Surabaya, Indonesia. A cross-sectional study was conducted, and 1066 students were evaluated as respondents. (3) Result: The results of multivariate analyses showed that following factors were associated with better health behavior: female, better academic performance, higher grade, higher father’s education, lower allowance, and better CHL and FHL. The strongest association for HB was gender. CHL was especially associated with handwashing, physical activity, and drug abuse. FHL was associated with smoking and drug abuse. The implementation of HPS programs did not reach an optimum level and only influenced physical activity. (4) Conclusions: The findings confirm that CHL and FHL have a significant association with several HBs. HB intervention in the HPS program is recommended to incorporate the CHL and FHL for a better health impact.

## 1. Introduction

Human behavior strongly related to health (i.e., health behavior; HB) is an important factor in public health because it influences individual health outcomes of both communicable and noncommunicable diseases (NCDs) [1,2]. Health behaviors concerning NCDs include smoking behavior, unhealthy diet, alcohol use, and physical inactivity, while those relating to communicable diseases include washing hands after using the toilet, covering when cough/sneezing, and wearing a face mask to reduce the spread of diseases [3,4,5]. Many health behaviors in adults that are beneficial or become a health risk were adopted during adolescence and have continued into adult life [6,7]. The habituation of better health behaviors in adolescence is a global imperative effort to decrease public health risks and to prevent poor individual health outcomes [8,9,10,11,12].

The formation of better HB in adolescents has also been a longstanding task for Indonesia. Indonesia is an upper-middle-income country [13] and the world’s largest archipelago nation, with a total population of 268 million people [14]. Indonesia experiences a double burden in health, as the country still lists communicable diseases in its top 10 causes of death in addition to NCDs [15]. To improve this situation, a change in health behavior in people living in the country is critically important, and adolescents (people from 10 to 19 years old), accounting for 16.92% of the national population [14], are expected to be an effective target for health-promoting interventions.

Adolescents’ health behavior problems have been recognized globally [16,17]. The Global School Health Survey (GSHS) 2015, administered by WHO and the Indonesian Ministry of Health, reported that Indonesian adolescents who studied at junior to senior high school had health behavior problems such as “not always washing hands with soap” (36.42% of 11,028 respondents), “high consumption on fast food” (54.38% of 11,046 respondents), “high intake of soda drink per day” (62.45% of 11,049 respondents), “less consumption of vegetables-fruits” (76.77% of 11,056 respondents), “lack of adequate physical activity” (46.84% of 10,880 respondents), and “emotional disturbance” (62.38% of 11,110 respondents) [18]. Although these adolescent health behavior problems are also commonly observed worldwide [16,19], they need considerable attention for the future improvement of health behavior in Indonesians. Hence, effective intervention in the adolescents’ health behavior problems should be provided.

Considering the improvement of adolescents’ HB, understanding the causes and the risk factors associated with the problems is important. Socioeconomic factors have been proved to be important for shaping health outcomes and HB in adolescents globally [20,21,22]. One study defined “children at risk” as children and adolescents with low social economic status that causes an impact on their health outcomes [23]. Other studies conducted in Europe (32 countries) and Latin American countries (Argentina and Mexico) showed that lower education levels of parents and lower family wealth conditions were considered as the factors that influenced alcohol and drug use in adolescents [24,25,26]. Those factors also influenced the academic performance of students, which, in turn, was considered to affect the adoption of other risky HBs such as smoking, lower physical activity, and lower diet quality [27,28]. The internet accessibility of students, as a result of the advance in information technology and the capacity of infrastructure in family settings, was also suggested as a significant factor for the formation of health behavior, either in a positive or negative way [29,30,31,32].

Health literacy (HL) is recognized widely as a strong factor for the health outcome and HBs in adolescence [33,34,35,36,37,38]. WHO defined HL as a person’s capacity to obtain and comprehend health information and services, as well as to use this information to make better health decisions [39]. Two types of HL are commonly used in scientific publications: functional and comprehensive health literacy [40,41]. Functional health literacy (FHL) refers to the personal ability to read and understand health-related information [40], while comprehensive health literacy (CHL) is the ability to seek information, understand, appraise, and apply it to make beneficial health choices [41]. Both types of HL are considered to be very important modifying factors that should be included in health promotion in the school setting, which is the best institution for adolescents at learning age to adopt important health life skills [42,43].

To intervene in health through health education and promotion at school, the Indonesian government established a health-promoting school (HPS) program in 1980 with collaboration from four ministries (Ministry of Education and Culture, Ministry of Health, Ministry of Religion, and Ministry of Home Affairs). This HPS program, called “Usaha Kesehatan Sekolah” (UKS), has the overall goal to raise student academic achievement by enhancing positive life skills through a healthy school atmosphere, health care, health education, and health behavioral change [44]. Studies have shown that the HPS program has a positive impact in increasing health knowledge and awareness, as well as instilling good HB practices [45,46,47]. However, implementation of HPS in Indonesia has met many challenges because of a lack of priority and funding, difficulty in coordinating a large number of schools and students, different levels of development among the regions, a very wide diversity of Indonesian cultures, the limited role of teachers and health personnel, and poor record-reporting systems [48,49]. These problems may be limitations on the effectiveness of the HPS, and hence, a clear understanding of the effectiveness of HPS is required.

Under the current situation in Indonesia, this study was guided by two questions: (i) What is the effect of health literacy on better health behavior in adolescents in the Indonesian school environment when adjusting the influence of socioeconomic factors?, and (ii) What is the impact of health promotion in Indonesian schools in the practice of better health behavior? To answer these questions, we measured the HL and HB in students of multiple high schools in Indonesia and quantified HPS in the schools. Then, we analyzed the cross-sectional association among HL, HPS, and HB by considering the influence of possible socioeconomic factors on the development of HB in adolescents.

## 2. Materials and Methods

### 2.1. Study Site and Population

Surabaya, the second-largest city in Indonesia, located in the East Java Province with an area of 326.81 km^2^ and 5 administrative regions, was selected as the study site. In 2019, the population of Surabaya reached 3.094 million, with adolescents accounting for 16.14% (499,862) of the total population. There are three types of high schools in Indonesia: academic high schools and vocational high schools regulated by the ministry of education, and Islamic high schools regulated by the ministry of religion. The total number of academic high schools was 141 (22 public schools, 119 private schools) in 2019, with 62,249 students (22,767 students in public schools, 39,482 students in private schools). The total number of vocational high schools was 103 (10 public and 93 private), with 63,048 students (21,459 public, 41,589 private). Lastly, the total number of Islamic High Schools was 10, with 5022 students. [50] Surabaya also ranks second in Regional Gross Domestic Product by cities in Indonesia with IDR 580.756 billion (USD 41.25 million). [51] In 2019, the total workforce reached 1.747 million, of which the largest composition was academic-vocational high school education (39.13%), while diploma-university education reached 18.01% [50].

### 2.2. Description of Survey

A cross-sectional study was conducted in Surabaya City with ethical approval from Hiroshima University (7 August 2019; approval register number E-1705) and permission from the Surabaya City education office (number 420/3795/101.6.25/2019). The participants were recruited using convenient cluster random sampling from high schools in five administrative regions of Surabaya City from mid-December 2019 to mid-January 2020. We first set an inclusion criterion of high schools following the characteristics of the school. The school’s categories used in the present study were general public, general private, and vocational high schools under the Ministry of Education. From five different administrative regions of Surabaya City, three high schools belonging to each category were then chosen using convenient sampling. From there, using cluster random sampling, two classes were selected from two different majors from the same grade. For the public school and private high school, one class was selected from the natural and social science major, and one class was selected from two different majors in the vocational high school.

The survey type was anonymous and voluntary, and the research team acquired informed consent from the parents and informed assent from the students. The self-administered questionnaire that included informed assent was given to the participants, while the study purpose and how to participate were explained. The participants in this study had an opportunity to ask a question during the data retrieval process or cancel participation before the questionnaire was collected. Two investigators guided them during the process.

### 2.3. Measures

#### 2.3.1. BMI and Health Behaviors

The GSHS Indonesian Questionnaire 2015 was used to measure health behavior using the primary cause of morbidity and mortality among children and adults worldwide [18]. The five HB used in this study were handwashing, physical activity, smoking, alcohol use, and drug abuse. BMI (Body Mass Index) was included as a health outcome that can reflect the nutritional diet. Every response was converted to a binomial scale by giving them a value of 1 if it has benefit to health and 0 if it compromises health.

BMI status was calculated from self-reported weight and height data of the respondents using the BMI formula and categorized using the WHO BMI score per age for boys and girls aged 5–19 [52]. BMI status was coded as 1: normal or 0: not normal (malnourished/overweight/obese). The handwashing variable was derived from the question, “During the past 30 days, how often did you use soap when washing your hands?” Five responses ranged from never to always, coded always (1) or never to most of the time (0). The physical activity variable was derived from the question, “During the past 7 days, how many days were you physically active for a total of at least 60 min per day? Add up all the time you spent in any kind of physical activity each day.” Eight answers ranged from 0 days to 7 days, coded 1: 5 to 7 days or 0: 0 to 4 days. Smoking was measured using the question, “During the past 30 days, how many days did you smoke cigarettes?” Seven responses ranged from 0 days to all 30 days, coded 1: 0 days or 0: 1 to all 30 days. Alcohol use was measured using the question, “During the past 30 days, how many days did you have at least one drink containing alcohol?” Seven answers ranged from 0 days to all 30 days, coded 1: 0 days or 0: 1 to all 30 days. Drug abuse was measured using the question, “During your life, how many times have you used amphetamines or methamphetamines (also called ecstasy)?” Five responses ranged from 0 times to 20 or more times, coded 1: 0 times or 0: 1 to 20 or more times.

#### 2.3.2. Comprehensive Health Literacy (CHL)

HLS-EU-16 is a short version of HLS-EU-47, which was designed to measure health literacy at a community level using 16 questions that measured people’s perceptions of their ability to discover, understand, judge, and apply health information to preserve and enhance their health [53]. Permission for using HLS-EU-Q16 was acquired by email from the European Health Literacy Project’s coordinator [54]. This self-reported instrument uses Likert-type responses (very easy, easy, difficult, and very difficult), and can be administered using a paper-pencil questionnaire, telephone, or internet form. For scoring HLS-EU-16, responses were converted into binary, coded 1 for “very easy” and “easy,” or 0 for “difficult” and “very difficult.” The response “don’t know” or a refusal to answer was counted as missing. The CHL score was calculated using the sum of all answers and could range from 0–16. Only respondents who answered a minimum of 14 questions were accounted for analysis. The total score was converted into 3 categories: “sufficient” for a score more than 12, “problematic” for a score from 9 to 12, and “inadequate” for a score less than 9, as recommended by Pelikan et al. [53].

#### 2.3.3. Functional Health Literacy (FHL)

The NVS (Newest Vital Sign) is an instrument that assesses people’s ability to read and understand information in a health context. Nutritional labels of ice cream and six-question items were used to assesses people’s ability to apply health knowledge to read and understand the information in words and numbers. In the study, the total number of correct answers was utilized, with equal or more than four correct answers indicating average literacy, two to three correct answers suggesting marginal literacy, and less than two correct answers indicating limited literacy [55].

#### 2.3.4. Socioeconomic Factors

Socioeconomic factors in this research were self-reported, and consisted of gender, grade, academic achievement, father’s education, mother’s education, allowance, and internet access.

#### 2.3.5. Indonesian HPS Program Instrument

The HPS Instrument, developed by the Indonesian Ministry of Health in 2010, was used to measure the implementation of HPS in high schools. This instrument consists of three parts that measure the implementation of health education, health service, and healthy school environment through a series of checklist requirements that must be confirmed by observation and interviews with the HPS manager. There are four levels of achievement: minimum (1), standard (2), optimum (3), and perfect (4), which can be achieved by the fulfillment of all the checklist requirements in that level. The minimum level can be granted even if the checklist is not fulfilled. The score from each part is combined into one score, which is categorized as minimum (1–3), standard (4–6), optimum (7–9), or perfect (>9). The Indonesian Instrument is shown in the Supplementary Materials.

#### 2.3.6. Statistical Analysis

Proportions were calculated from socioeconomic variables, which consisted of gender, academic performance, father’s education, mother’s education, allowance, and internet access. Every category of variables was constructed using conceptual theoretical consideration, which made the number of categories in each variable vary from 2 to 4. Cross tabulation was used to describe the distribution of health-promoting school, health literacy, and health behavior in the school context, while the Chi-square test was used to check the association between two variables.

Statistical analyses were performed using IBM SPSS Statistics for Windows version 25 (IBM, Armonk, NY, USA). For each analysis, an alpha level of 0.05 was considered significant. Associations between socioeconomic variables, HPS, CHL, FHL, five HBs, and BMI among high school students were evaluated, and the crude odds ratios were calculated (i.e., bivariate analysis). Two binomial logistic regression models, one using CHL and the other using FHL, were constructed to evaluate the influence to each response variable (i.e., one of the five HBs or BMI) from predictors (i.e., sociodemographic factors HPS, and CHL or FHL) using the backward elimination method.

## 3. Results

### 3.1. Respondent Characteristic

From the 15 high schools involved in this study, 1066 students participated. After processing the data, 106 respondents (10.94%) were excluded because they did not fulfill the criteria of inclusion on the HLS-EU-Q16 Questionnaire. The characteristics of the study participants are presented in Table 1. Among 960 students who participated, 591 (61.56%) were female. Participants were aged from 14 to 19 years old, with a mean age of 16.19 years old, and the majority of the age group were 16 years old (53.33%). Most students’ academic performance was at a high level of 74.69%, with no difference between female and male students. Father’s education and mother’s education had a similar trend: secondary education accounted for the largest proportion (54.06% of fathers, 54.69% of mothers); while higher education comprised the second-largest proportion (35.94% of fathers, 31.25% of mothers). In general, father’s education was slightly higher than mother’s education. For economic status measured by students’ monthly allowance, the majority of students (77.92%) had middle and low economic status (<USD 35). For internet access, most of the students had private internet. However, 26.88% did not have internet access. Health-promoting school programs in 15 schools in this study were considered inadequate, with 63.85% of respondents assigned to the minimum HPS level. Regarding CHL, 64.27% of respondents reported sufficient CHL, while for FHL, only 25.94% had average literacy. There was a significant difference in FHL (*p* < 0.001) between female and male students. Female students showed better behavior regarding smoking, alcohol use, drug abuse, and BMI. The prevalence of health risk behavior such as smoking, alcohol use, and drug abuse were very low, i.e., only 12.50%, 9.06%, and 1.46%, respectively. Handwashing among students was considered insufficient, because only 463 students (48.23%) always used soap. The number of high school students with sufficient physical activity per week was only 256 (26.67%), which requires further consideration.

### 3.2. HPS Association with CHL, FHL, and HBs

Chi-square analysis was used to examine the association of HPS on CHL, FHL, and HBs (Table 2). The results showed that HPS had a significant association with CHL (*p* <0.01), FHL (*p* < 0.001), and physical activity (*p* < 0.01). In the CHL and physical activity variables, the standard HPS level showed better results compared with the minimum level. On the contrary, in FHL, the minimum HPS level had a better result.

### 3.3. The Relationship between Each Health Behaviors and Student Socioeconomic Factors, Health Promoting School, CHL, and FHL

For BMI (in Table 3), in the univariate regression analyses, female gender, grade 10, and adequate FHL were associated with better BMI. Multivariate model for CHL and FHL produced the same model, which retained the female gender (odds ratio, CI 95%; 0.5, 0.4–0.6), father’s education (5.2, 1.1–23.7) and mother’s education (0.2, 0.04–1.1) as factors associated with better BMI.

In terms of handwashing (in Table 4), in the univariate analysis, high academic performance, unlimited internet access, and CHL were associated with better handwashing. In multivariate model 1 (CHL), the highest academic performance (odds ratio, CI 95%; 2.2, 1.3–3.6) and sufficient CHL (1.9, 1.1–3.6) coincided with better handwashing. For the FHL model, only academic performance (2.2, 1.3–3.7) was associated with better handwashing.

For physical activity (in Table 5), in the univariate analyses, grade 12, highest academic performance, high internet access, and minimum HPS were correlated with greater physical activity in a week. In multivariate model 1 (CHL), female gender (odds ratio, CI 95%; 0.7, 0.6–1.0), grade 12 (1.7, 1.1–2.7), sufficient HL (1.8, 0.8–3.7), and standard HPS (0.5, 0.4–0.8) were associated with greater physical activity. In multivariate model 2 (FHL), grade 12 (1.8, 1.1–2.8), highest academic performance (2.1, 1.1–3.9), and standard HPS (0.6, 0.4–0.9) were also associated with greater physical activity.

In terms of smoking (In Table 6), in the univariate analysis, female gender, high academic performance, higher father’s education, high allowance, problematic HL, and adequate FHL were associated with no smoking behavior. In multivariate model 1 (CHL), female gender (odds ratio, CI 95%; 0.1, 0.1–0.1), high academic performance (2.6, 1.4–4.6), primary father’s education (13.1, 1.9–90.5), primary mother’s education (0.3, 0.04–1.6) and high allowance (0.5, 0.3–0.8) contributed to no smoking behavior. For multivariate model 2 (FHL), female gender (odds ratio, 0.1, 0.1–0.2), high academic performance (2.6, 1.4–4.7), primary father’s education (11.3, 1.6–79.2), primary mother’s education (0.2, 0.04–1.4), high allowance (0.4, 0.2–0.7), and adequate FHL (6.8, 2.9–15.9) contributed to no smoking behavior.

For alcohol use (in Table 7), in the univariate analyses, female gender, high academic performance, higher father’s education, low allowance, and adequate FHL were associated with no alcohol use behavior. In multivariate model 1 (CHL), female gender (odds ratio, CI 95%; 0.3, 0.2–0.4), high academic performance (2.4, 1.2–4.4), higher father’s education (9.0, 2.1–38.4), and low allowance (0.3, 0.2–0.5) contributed to no alcohol use behavior. In multivariate model 2 (FHL), female gender (odds ratio, 0.3, 0.2–0.4), high academic performance (2.3, 1.2–4.4), higher father’s education (7.4, 1.7–33.1), low allowance (0.2, 0.1–0.5), and low FHL (2.2, 1.3–3.9) contributed to no alcohol use behavior.

In terms of drug abuse (in Table 8), in the univariate analysis, female gender, sufficient HL, and adequate FHL were associated with no drug abuse. In multivariate model 1 (CHL), female gender (odds ratio, CI 95%; 0.1, 0.0–0.4), sufficient HL (9.3, 2.1–41.3), and minimum HPS (0.3, 0.1–0.9) contributed to no drug abuse. For model 2 (FHL), female gender (odds ratio, 0.1, 0.0–0.6) and marginal FHL (12.8, 1.7–99.8) contributed to no drug abuse.

## 4. Discussion

Focusing on BMI and five health behaviors, the present study measured the associations between the adolescents’ BMI, health behaviors, and health literacy in the Indonesian high school environment, considering the influence of health promotion in their high schools. Health promotion in high schools showed a significant positive association with CHL status, but a negative association with FHL status and with the status of physical activity. In the logistic regression analyses between the health behaviors and either of the two types of health literacy (i.e., CHL and FHL), taking the socioeconomic characteristics into account, better status of the health literacy was not always a significant factor for predicting better health behavior. However, we found that health literacy above the lowest status (inadequate in CHL and limited in FHL) was positively associated with better HB. CHL is possibly associated with a positive effect on handwashing behavior, physical activity behavior, and drug abuse behavior, whereas FHL is possibly associated with a positive effect on smoking, alcohol use, and drug abuse. Based on the model that explains the mechanism linking HL to behavior and health status proposed by Osborn et al. (2011), desirable HB and health outcomes will not be achieved if HL cannot improve knowledge and build self-efficacy [56].

We implemented two types of health literacy instruments that were already validated and used internationally to gain more comprehensive knowledge. The first is the HLS-EU-16 questionnaire, which measures CHL using respondent perception scores on health literacy skills (i.e., finding, understanding, judging, and applying the information) about health care, promotion, and disease prevention. The second is NVS, which measures FHL by applying tests for literacy and numeracy on health information. The difference between these two measurements has already been recognized widely, yet there has been no comprehensive use of these measurements to study the situation in Indonesia. We found that the FHL of high school students in Surabaya was quite worrying, because only 249 (25.9%) of students reached the average health literacy level of the ability to read and understand health information. A possible explanation for this phenomenon is the low level of reading literacy and mathematical ability of Indonesian students. Their scores, as measured in the Programme for International Student Assessment (PISA) 2018, were below the average of OECD countries [57]. A different result was shown for CHL, which measured students’ perception in finding, understanding, judging, and applying health information in a health setting. As to CHL, 617 students (64.37%) reached a sufficient level. The gap between students’ FHL and CHL shows the existence of a problem in HL that needs to be resolved.

In the present study, we confirmed the difference in the contribution of CHL and FHL to HB. CHL was significantly associated with handwashing and physical activity, while FHL was significantly associated with smoking and alcohol use. The only response variable associated with both CHL and FHL was drug abuse. This observation is understandable, because a “decision-making process” closely related to CHL is required to enhance handwashing behavior and to improve physical activity. The result of CHL association with handwashing behavior was also shown in previous studies in older adults in Hong Kong [58], adolescents in Norway [59], and intensive care unit visitors in Thailand [60]. Generally, a positive association between physical activity and HL has been shown by previous studies in many countries, as listed in the systematic review by Buja (2020). From 22 studies included in the systematic review, 18 showed a positive association, but 4 studies conducted by Al Sayah et al. (2012), Lee (2012), Mitsutake (2012), and Wolf (1997) reported no association. Among these four studies, only Mitsutake (2012) used CHL, while the other three researchers used FHL [61]. The same results about the association between smoking behavior and FHL were found by two studies conducted in the USA by Stewart et al. (2013) and Marie et al. (2014), but a study in Guatemala by Hoffman et al. (2017) produced different results [62,63,64]. Studies by Chisolm et al. (2014), Hoffman et al. (2017), and Amoah et al. (2019) confirmed that alcohol consumption is affected by FHL [35,64,65]. For the association between HL and drug abuse, we cannot find any previous study in any journal database. FHL reflects the ability to understand health information. Smoking, alcohol use, and drug abuse are behaviors that involve directly intaking harmful substances, and these behaviors are connected to immediate health risks. This means that the “decision-making process” connecting CHL was possibly not necessary in those health behaviors associated with FHL.

In contrast to the health behaviors related to FHL or CHL, the association between BMI and health literacy was not clear in the present study. Although average FHL (odds ratio, CI 95%; 1.494, 1.027–2.174) had an association with BMI in the univariate analysis, BMI was associated with gender, grade, and father’s education, but not with CHL nor FHL in the multivariate analysis. The study results on the association between FHL and BMI are different from those of Chari et al. (2014), who targeted children and adolescents in the United States (US) and concluded that there was a strong association after adjusting with other variables [66]. This is consistent with the observation in many countries listed in a systematic review about adult health behavior derived from Saudi Arabia, the USA, Netherlands, Australia, and Scotland [67]. However, some studies have reported a similar observation with our results, with no association between adult BMI and CHL in Japan [68], China (Liu et al., 2015), Hawaii (Sentell et al., 2011), and Iran [69], and no association between BMI and FHL in the USA (Wolf et al., 2007; Lanpher et al., 2016). The situation in Indonesia adds additional evidence for the lack of association between BMI and CHL/FHL. To clarify the effect of CHL/FHL on BMI, further study with a concrete design (e.g., experimental study or longitudinal study) is required.

We found that HPS implementation at the Indonesian high school was not associated strongly with HB, CHL, and FHL. In the multivariate regression model, HPS was negatively associated with the physical activity behavior and drug abuse in the CHL model, and negatively associated with physical activity in the FHL model. In a HB model using CHL, HPS could be retained, but this result was not found in the HB model using FHL. For HPS implementation, we found that only five schools (33.33%) achieved a standard level in Indonesian HPS measurement, while the others only reached the minimum level (Supplementary Material). This indicates that the implementation of health promotion in schools has not achieved satisfactory results. From observations of the facilities and interviews with the HPS managers, we found the implementation of HPS is very dependent on funding, awareness of the importance of the health aspects of students by the principal, understanding of the implementation of the HPS system by schools, guidance from community health centers to schools, and the involvement of the physical education (PE) teacher in teaching health education (data not shown). As a result, the delivery of health promotion is different among schools, and the contribution of health promotion to students’ health literacy and health behavior may be difficult to measure. The future improvement of health promotion and intervention in schools (to a satisfactory level) should be designed using the best practices from previous studies [70,71], and further analysis should conducted on the relationship between HPS, HL, and HB.

Among socioeconomic factors, gender had the strongest association with health behaviors, followed by academic performance and father’s education. Male gender was negatively associated in both CHL and FHL models with BMI, smoking, alcohol use, and drug abuse, and it was also negatively associated with physical activity in the CHL model. These study results are similar to previous studies in the USA, European countries, Denmark, and Greece showing that, in general, females report better health behavior in oral hygiene, diet, alcohol use, substance abuse, reproductive health, and BMI, and males only score better than females in physical activity [72,73,74,75]. The difference in health behavior in adolescent females and males is caused by the biological factor, the different context of social roles, body expression for sexuality, influence from the social environment, and interaction with the health care system [73]. Academic performance had a positive impact on handwashing, physical activity, smoking, and alcohol use in both CHL and FHL models. These results confirm the previous findings that academic achievement has a positive influence toward health behavior [27,28,76,77].

Health promotion, or the improvement of health behaviors through advanced health literacy, must be considered in relation to internet use, since information and communication technology (ICT) has been developing and prevailing very rapidly. In our survey, the availability of high-speed internet tended to contribute to good health literacy and behaviors. The use of ICT, for example, through the utilization of social media by the health education staff of schools, can be expected to contribute to forthcoming health promotion activities. The HPS manager’s ability to design study material on the internet as a source of health information may also be helpful in using the principle of health literacy skill to empower students to gain health benefits.

This research has several limitations that should be considered for future research. First, although the sample size was adequate to fulfill the goal of the study, it was still considered too small to represent the wide range of Indonesian population characteristics such as ethnicity, as well as socioeconomic and developmental progress in the region. Second, CHL, FHL, and HB were all measured with self-reporting questionnaires, and these questionnaires may have some respondents to report responses better than their actual status to make them more socially acceptable. In addition, self-reporting questionnaires may have led to inaccurate interpretations of the question by the respondents. To increase honesty and reduce this socially acceptable response tendency, we used anonymity and guaranteed privacy of the data in the informed consent statement that was read and explained by investigators before respondents self-filled the questionnaire. To lessen incorrect interpretation of questions in the instrument, investigators guided and answered questions while students filled out the questionnaire. Finally, because of our study’s cross-sectional nature, we were unable to establish if there was a causal association between health behavior and health literacy, health-promoting school programs, and others socioeconomic factors. A longitudinal study may be useful in resolving this issue.

## 5. Conclusions

The study confirmed the importance of health literacy (CHL and FHL) which showed a strong relationship with health behavior (HB). Overall, better health literacy led to better HB. The results also showed that socioeconomic factors had both positive and negative impacts on HB. The positive impact was demonstrated by the female gender, the student’s academic performance, and father’s education, whereas negative impact was shown by the student’s monthly allowance. Moreover, the HPS program, as an effort to increase health knowledge and awareness of students in school in Indonesia, had no strong association with HB. The study result can be used as a baseline in understanding the importance of health literacy on health behavior in the health-promoting school setting. Based on our results, to deliver effective health promotion in the school environment, the HPS intervention should incorporate health literacy skills specifically tailored to the student’s socioeconomic conditions. The HPS should also involve the student’s families, as well as the community in the school area. Social media can be utilized by physical education teachers or HPS managers in the delivery of the health promotion and intervention to students and parents to strengthen the HPS program.

## Figures and Tables

**Table 1:** Socioeconomic characteristics of high school students in Surabaya, Indonesia.

Variables	Female	Male	Total	*p*-Value ^1^
Grade				
10	216 (64.5%)	119 (35.5%)	335 (34.90%)	0.008 **
11	263 (56.8%)	200 (43.2%)	463 (48.23%)	
12	112 (69.1%)	50 (30.9%)	162 (16.88%)	
Academic Performance				
Middle-Low	59 (55.1%)	48 (44.9%)	107 (11.15%)	0.302
High	450 (62.8%)	267 (37.2%)	717 (74.69%)	
Highest	82 (60.3%)	54 (39.7%)	136 (14.17%)	
Father’s Education				
No Formal Education	6 (60.0%)	4 (40.0%)	10 (1.04%)	0.014 *
Primary Education	66 (76.7%)	20 (23.3%)	86 (8.96%)	
Secondary Education	320 (61.7%)	199 (38.3%)	519 (54.06%)	
Higher Education	199 (57.7%)	146 (42.3%)	345 (35.94%)	
Mother’s Education				
No Formal Education	6 (42.9%)	8 (57.1%)	14 (1.46%)	0.023 *
Primary Education	86 (71.1%)	35 (28.9%)	121 (12.60%)	
Secondary Education	328 (62.5%)	197 (37.5%)	525 (54.69%)	
Higher Education	171 (57.0%)	129 (43.0%)	300 (31.25%)	
Allowance				
Low	197 (58.6%)	139 (41.4%)	336 (35.00%)	0.014 *
Middle	275 (66.7%)	137 (33.3%)	412 (42.92%)	
High	119 (56.1%)	93 (43.9%)	212 (22.08%)	
Internet Access				
No Private Internet	167 (64.7%)	91 (35.3%)	258 (26.88%)	0.077
<11 Giga	130 (55.6%)	104 (44.4%)	234 (24.38%)	
>11G, <Unlimited	116 (59.2%)	80 (40.8%)	196 (20.42%)	
Unlimited	178 (65.4%)	94 (34.6%)	272 (28.33%)	
CHL				
Inadequate	34 (66.7%)	17 (33.3%)	51 (5.31%)	0.201
Problematic	190 (65.1%)	102 (34.9%)	292 (30.42%)	
Sufficient	367 (59.5%)	250 (40.5%)	617 (64.27%)	
FHL				
Limited	174 (52.7%)	156 (47.3%)	330 (34.38%)	0.000 ***
Marginal	230 (60.4%)	151 (39.6%)	381 (39.69%)	
Average	187 (75.1%)	62 (24.9%)	249 (25.94%)	
Health Promoting School				
Minimum	357 (58.2%)	256 (41.8%)	613 (63.85%)	0.005 ***
Standard	234 (67.4%)	113 (32.6%)	347 (36.15%)	
BMI				
Not Normal	129 (48.3%)	138 (51.7%)	267 (100.0%)	0.000 ***
Normal	462 (66.7%)	231 (33.3%)	693 (100.0%)	
Health Behavior				
Handwashing				
Never to most of time	301 (60.6%)	196 (39.4%)	497 (51.77%)	0.510
Always	290 (62.6%)	173 (37.4%)	463 (48.23%)	
Physical activity				
Less than 5	425 (60.4%)	279 (39.6%)	704 (73.33%)	0.208
5 to 7 days	166 (64.8%)	90 (35.2%)	256 (26.67%)	
Smoking				
1 or 2 to all 30 day	19 (15.8%)	101 (84.2%)	120 (12.50%)	0.000 ***
0 day	572 (68.1%)	268 (31.9%)	840 (87.50%)	
Alcohol use				
Consume Alcohol	27 (31.0%)	60 (69.0%)	87 (9.06%)	0.000 ***
No Alcohol	564 (64.6%)	309 (35.4%)	873 (90.94%)	
Drugs Abuse				
1–10 or more times	2 (14.3%)	12 (85.7%)	14 (1.46%)	0.000 ***
0 Times	589 (62.3%)	357 (37.7%)	946 (98.54%)	

^1^ Chi-Square test; ***: *p* < 0.001; **: *p* < 0.01; *: *p* < 0.05.

**Table 2:** Associations between health-promoting school with CHL, FHL dan health behaviors.

Variables	Health-Promoting School		*p*-Value ^1^
	Minimum	Standard	
CHL			
Inadequate	39 (6.36%)	12 (3.46%)	0.008 **
Problematic	201 (32.79%)	91 (26.22%)	
Sufficient	373 (60.85%)	244 (70.32%)	
FHL			
Limited	184 (30.02%)	146 (42.07%)	0.000 ***
Marginal	249 (40.62%)	132 (38.04%)	
Average	180 (29.36%)	69 (19.88%)	
BMI			
Not Normal	169 (27.57%)	98 (28.24%)	0.823
Normal	444 (72.43%)	249 (71.76%)	
Health Behavior			
Handwashing			
Never to most of time	321 (52.37%)	176 (50.72%)	0.624
Always	292 (47.63%)	171 (49.28%)	
Physical activity			
Less than 5 days	427 (69.66%)	277 (79.83%)	0.001 **
5 to 7 days	186 (30.34%)	70 (20.17%)	
Smoking			
1 or 2 to all 30 day	85 (13.87%)	35 (10.09%)	0.089
0 day	528 (86.13%)	312 (89.91%)	
Alcohol use			
Consume Alcohol	51 (8.32%)	36 (10.37%)	0.287
No Alcohol	562 (91.68%)	311 (89.63%)	
Drugs Abuse			
2–10 or more times	6 (0.98%)	8 (2.31%)	0.099
0 Times	607 (99.02%)	339 (97.69%)	

^1^ Chi-Square test; ***: *p* < 0.001; **: *p* < 0.01.

**Table 3:** Univariate and multivariate logistic regression of BMI with socioeconomic factors in the CHL and FHL models.

Variables	BMI					
	Univariate		Model 1 (CHL) Pseudo R^2^ 0.080		Model 2 (FHL) Pseudo R^2^ 0.080	
	COR (CI 95%)	*p*	AOR (CI 95%)	*p*	AOR (CI 95%)	*p*
Gender						
Female	ref.		ref.		ref.	
Male	0.467 (0.351–0.623)	0.000 ***	0.415 (0.308–0.560)	0.000 ***	0.415 (0.308–0.560)	0.000 ***
Grade						
10	ref.		ref.		ref.	
11	0.910 (0.659–1.257)	0.569	0.864 (0.605–1.234)	0.422	0.864 (0.605–1.234)	0.422
12	0.545 (0.364–0.816)	0.003 ***	0.437 (0.281–0.680)	0.000 ***	0.437 (0.281–0.680)	0.000 ***
Academic Performance						
Middle-Low	ref.		-		-	
High	1.292 (0.836–1.998)	0.249	-		-	
Highest	1.350 (0.775–2.353)	0.289	-		-	
Father Education						
No Formal Education	ref.		ref.		ref.	
Primary Education	2.071 (0.554–7.747)	0.279	3.645 (0.752–17.672)	0.108	3.645 (0.752–17.672)	0.108
Secondary Education	2.873 (0.819–10.079)	0.099	5.126 (1.106–23.751)	0.037 *	5.126 (1.106–23.751)	0.037 *
Higher Education	2.450 (0.694–8.648)	0.164	3.818 (0.794–18.349)	0.094	3.818 (0.794–18.349)	0.094
Mother Education						
No Formal Education	ref.		ref.		ref.	
Primary Education	0.532 (0.141–2.014)	0.353	0.202 (0.039–1.048)	0.057	0.202 (0.039–1.048)	0.057
Secondary Education	0.715 (0.197–2.599)	0.610	0.295 (0.058–1.497)	0.141	0.295 (0.058–1.497)	0.141
Higher Education	0.776 (0.211–2.855)	0.703	0.440 (0.084–2.309)	0.332	0.440 (0.084–2.309)	0.332
Allowance						
Low	ref.		-		-	
Middle	0.928 (0.670–1.284)	0.650	-		-	
High	0.802 (0.549–1.173)	0.256	-		-	
Internet Access						
No Private Internet	ref.		-		-	
<11 Giga	0.822 (0.551–1.227)	0.338	-		-	
>11G, <Unlimited	0.680 (0.451–1.026)	0.066	-		-	
Unlimited	0.916 (0.620–1.354)	0.661	-		-	
CHL						
Inadequate	ref.		-			
Problematic	1.206 (0.625–2.326)	0.577	-			
Sufficient	1.036 (0.553–1.939)	0.913	-			
FHL						
Limited	ref.				-	
Marginal	1.177 (0.852–1.626)	0.323			-	
Average	1.494 (1.027–2.174)	0.036			-	
HPS						
Minimum	ref.		-		-	
Standard	0.967 (0.721–1.297)	0.823	-		-	

COR: Crude Odd Ratio; AOR: Adjusted Odd Ratio; ***: *p* < 0.001; *: *p* < 0.05; Grey background: the predictor variable not included in analysis.

**Table 4:** Univariate and multivariate logistic regression of handwashing with socioeconomic factors in the CHL and FHL models.

Variables	Handwashing					
	Univariate		Model 1 (CHL) Pseudo R ^2^ 0.027		Model 2 (FHL) Pseudo R ^2^ 0.013	
	COR (CI 95%)	*p*	AOR (CI 95%)	*p*	AOR (CI 95%)	*p*
Gender						
Female	ref.		-		-	
Male	0.916 (0.706–1.189)	0.510	-		-	
Grade						
10	ref.		-		-	
11	1.050 (0.792–1.391)	0.734	-		-	
12	1.120 (0.770–1.630)	0.553	-		-	
Academic Performance						
Middle-Low	ref.		ref.		ref.	
High	1.476 (0.974–2.239)	0.067	1.421 (0.934–2.161)	0.101	1.476 (0.974–2.239)	0.067
Highest	2.231 (1.330–3.744)	0.002 **	2.148 (1.275–3.617)	0.004 **	2.231 (1.330–3.744)	0.002 **
Father Education						
No Formal Education	ref.		-		-	
Primary Education	0.981 (0.258–3.734)	0.977	-		-	
Secondary Education	1.483 (0.414–5.316)	0.545	-		-	
Higher Education	1.407 (0.390–5.075)	0.602	-		-	
Mother Education						
No Formal Education	ref.		-		-	
Primary Education	0.833 (0.275–2.521)	0.747	-		-	
Secondary Education	0.882 (0.305–2.549)	0.816	-		-	
Higher Education	1.069 (0.366–3.122)	0.903	-		-	
Allowance						
Low	ref.		-		-	
Middle	1.085 (0.813–1.449)	0.579	-		-	
High	1.391 (0.985–1.964)	0.061	-		-	
Internet Access						
No Private Internet	ref.		-		-	
<11 Giga	1.324 (0.928–1.890)	0.121	-		-	
>11G, <Unlimited	1.220 (0.840–1.773)	0.295	-		-	
Unlimited	1.425 (1.012–2.008)	0.043 *	-		-	
CHL						
Inadequate	ref.		ref.			
Problematic	1.353 (0.728–2.514)	0.339	1.389 (0.743–2.597)	0.304		
Sufficient	1.988 (1.096 - 3.607)	0.024 *	1.992 (1.091–3.635)	0.025 *		
FHL					-	
Limited	ref.				-	
Marginal	0.919 (0.684–1.234)	0.575			-	
Average	0.996 (0.717–1.384)	0.982			-	
HPS						
Minimum	ref.		-		-	
Standard	1.068 (0.821–1.390)	0.624	-		-	

COR: Crude Odd Ratio; AOR: Adjusted Odd Ratio; **: *p* < 0.01; *: *p* < 0.05; Grey background: the predictor variable not included in analysis.

**Table 5:** Univariate and multivariate logistic regression of physical activity with socioeconomic factors in the CHL and FHL models.

Variables	Physical Activity					
	Univariate		Model 1 (CHL) Pseudo R ^2^ 0.058		Model 2 (FHL) Pseudo R ^2^ 0.042	
	COR (CI 95%)	*p*	AOR (CI 95%)	*p*	AOR (CI 95%)	*p*
Gender						
Female	ref.		ref.		-	
Male	0.826 (0.613–1.112)	0.208	0.758 (0.557–1.030)	0.076	-	
Grade						
10	ref.		ref.		ref.	
11	1.913 (1.369–2.673)	0.000 ***	1.633 (1.145–2.328)	0.007 **	1.629 (1.145–2.318)	0.007 **
12	1.598 (1.031–2.475)	0.036 *	1.711 (1.086–2.696)	0.021 *	1.769 (1.126–2.779)	0.013 *
Academic Performance						
Middle-Low	ref.		ref.		ref.	
High	1.602 (0.959–2.677)	0.072	1.479 (0.877–2.495)	0.142	1.562 (0.929–2.625)	0.092
Highest	2.011 (1.098–3.686)	0.024 *	2.001 (1.078–3.712)	0.028 *	2.106 (1.140–3.892)	0.017 *
Father Education						
No Formal Education	ref.		-		-	
Primary Education	2.217 (0.263–18.716)	0.464	-		-	
Secondary Education	3.489 (0.438–27.787)	0.238	-		-	
Higher Education	3.321 (0.415–26.576)	0.258	-		-	
Mother Education						
No Formal Education	ref.		-		-	
Primary Education	1.260 (0.262–6.051)	0.773	-		-	
Secondary Education	2.289 (0.506–10.355)	0.282	-		-	
Higher Education	2.491 (0.546–11.358)	0.239	-		-	
Allowance						
Low	ref.		-		-	
Middle	1.199 (0.866–1.661)	0.274	-		-	
High	0.960 (0.645–1.429)	0.839	-		-	
Internet Access						
No Private Internet	ref.		-		-	
<11 Giga	1.242 (0.827–1.867)	0.297	-		-	
>11G, <Unlimited	1.527 (1.006–2.317)	0.047 *	-		-	
Unlimited	1.144 (0.769–1.700)	0.507	-		-	
CHL						
Inadequate	ref.		ref.			
Problematic	1.174 (0.558–2.471)	0.673	1.190 (0.558–2.541)	0.653		
Sufficient	1.702 (0.835–3.471)	0.144	1.822 (0.880–3.772)	0.106		
FHL						
Limited	ref.				-	
Marginal	1.142 (0.814–1.603)	0.441			-	
Average	1.321 (0.912–1.914)	0.140			-	
HPS						
Minimum	ref.		ref.		ref.	
Standard	0.580 (0.424–0.794)	0.001 **	0.560 (0.390–0.805)	0.002 **	0.600 (0.419–0.858)	0.005 **

COR: Crude Odd Ratio; AOR: Adjusted Odd Ratio; ***: *p* < 0.001; **: *p* < 0.01; *: *p* < 0.05; Grey background: the predictor variable not included in analysis.

**Table 6:** Univariate and multivariate logistic regression of smoking with socioeconomic factors in the CHL and FHL models.

Variables	Smoking					
	Univariate		Model 1 (CHL) Pseudo R ^2^ 0.288		Model 2 (FHL) Pseudo R ^2^ 0.333	
	COR (CI 95%)	*p*	AOR (CI 95%)	*p*	AOR (CI 95%)	*p*
Gender						
Female	ref.		ref.		ref.	
Male	0.088 (0.053–0.147)	0.000 ***	0.078 (0.046–0.134)	0.000 ***	0.086 (0.050–0.148)	0.000 ***
Grade						
10	ref.		-		-	
11	1.175 (0.770–1.795)	0.455	-		-	
12	1.042 (0.598–1.816)	0.885	-		-	
Academic Performance						
Middle-Low	ref.		ref.		ref.	
High	2.832 (1.711–4.686)	0.000 ***	2.564 (1.440–4.565)	0.001 **	2.602 (1.440–4.699)	0.002 **
Highest	1.758 (0.925–3.339)	0.085	1.882 (0.900–3.938)	0.093	2.287 (1.071–4.885)	0.033 *
Father Education						
No Formal Education	ref.		ref.		ref.	
Primary Education	10.800 (2.282–51.111)	0.003 **	13.116 (1.901–90.508)	0.009 **	11.344 (1.626–79.156)	0.014 *
Secondary Education	4.009 (1.105–14.547)	0.035 *	5.110 (0.913–28.590)	0.063	4.067 (0.721–22.955)	0.112
Higher Education	5.550 (1.497–20.574)	0.010 *	5.349 (0.895–31.982)	0.066	4.242 (0.704–25.555)	0.115
Mother Education						
No Formal Education	ref.		ref.		ref.	
Primary Education	1.561 (0.396–6.149)	0.525	0.261 (0.043–1.596)	0.146	0.224 (0.036–1.377)	0.106
Secondary Education	1.744 (0.475–6.405)	0.402	0.514 (0.087–3.028)	0.462	0.411 (0.070–2.424)	0.326
Higher Education	2.649 (0.698–10.063)	0.152	0.941 (0.151–5.881)	0.948	0.704 (0.112–4.410)	0.708
Allowance						
Low	ref.		ref.		ref.	
Middle	0.882 (0.556–1.399)	0.593	0.702 (0.422–1.167)	0.172	0.626 (0.372–1.055)	0.079
High	0.550 (0.334–0.905)	0.019 *	0.449 (0.256–0.788)	0.005 **	0.361 (0.202–0.648)	0.001 **
Internet Access						
No Private Internet	ref.		-		-	
<11 Giga	1.096 (0.656–1.833)	0.726	-		-	
>11G, <Unlimited	1.395 (0.788–2.470)	0.253	-		-	
Unlimited	1.302 (0.781–2.170)	0.312	-		-	
CHL						
Inadequate	ref.		-			
Problematic	2.158 (1.010–4.612)	0.047 *	-			
Sufficient	1.958 (0.963–3.978)	0.063	-			
FHL						
Limited	ref.				ref.	
Marginal	1.855 (1.233–2.792)	0.003 **			1.832 (1.149–2.920)	0.011 *
Average	8.807 (3.966–19.559)	0.000 ***			6.774 (2.888–15.889)	0.000 ***
HPS						
Minimum	ref.		-		-	
Standard	1.435 (0.945–2.179)	0.090	-		-	

COR: Crude Odd Ratio; AOR: Adjusted Odd Ratio; ***: *p* < 0.001; **: *p* < 0.01; *: *p* < 0.05; Grey background: the predictor variable not included in analysis.

**Table 7:** Univariate and multivariate logistic regression of alcohol use with socioeconomic factors in the CHL and FHL models.

Variables	Alcohol Use					
	Univariate		Model 1 (CHL) Pseudo R^2^ 0.179		Model 2 (FHL) Pseudo R^2^ 0.197	
	COR (CI 95%)	*p*	AOR (CI 95%)	*p*	AOR (CI 95%)	*p*
Gender						
Female	ref.		ref.		ref.	
Male	0.247 (0.153–0.396)	0.000 ***	0.247 (0.150–0.406)	0.000 ***	0.257 (0.155–0.427)	0.000 ***
Grade						
10	ref.		-		-	
11	0.758 (0.462–1.242)	0.271	-		-	
12	1.096 (0.540–2.223)	0.800	-		-	
Academic Performance						
Middle-Low	ref.		ref.		ref.	
High	2.467 (1.365–4.456)	0.003 **	2.351 (1.243–4.447)	0.009 **	2.291 (1.205–4.355)	0.011 *
Highest	1.163 (0.572–2.365)	0.676	1.477 (0.680–3.207)	0.324	1.622 (0.738–3.562)	0.228
Father Education						
No Formal Education	ref.		ref.		ref.	
Primary Education	16.200 (3.495–75.093)	0.000 ***	10.541 (1.996–55.679)	0.006 **	9.284 (1.689–51.046)	0.010 *
Secondary Education	11.975 (3.327–43.107)	0.000 ***	11.372 (2.725–47.456)	0.001 **	8.959 (2.043–39.282)	0.004 **
Higher Education	8.324 (2.301–30.109)	0.001 **	9.040 (2.129–38.386)	0.003 **	7.387 (1.650–33.075)	0.009 **
Mother Education						
No Formal Education	ref.		-		-	
Primary Education	3.394 (0.799–14.411)	0.098	-		-	
Secondary Education	2.909 (0.783–10.810)	0.111	-		-	
Higher Education	2.455 (0.648–9.292)	0.186	-		-	
Allowance						
Low	ref.		ref.		ref.	
Middle	0.990 (0.546–1.793)	0.973	0.878 (0.474–1.628)	0.680	0.881 (0.473–1.641)	0.689
High	0.287 (0.164–0.502)	0.000 ***	0.275 (0.152–0.499)	0.000***	0.247 (0.135–0.453)	0.000 ***
Internet Access						
No Private Internet	ref.		-		-	
<11 Giga	1.073 (0.548–2.101)	0.838	-		-	
>11G, <Unlimited	0.665 (0.352–1.256)	0.208	-		-	
Unlimited	0.732 (0.402–1.336)	0.310	-		-	
CHL						
Inadequate	ref.		-			
Problematic	1.952 (0.787–4.842)	0.149	-			
Sufficient	1.533 (0.661–3.559)	0.320	-			
FHL						
Limited	ref.				ref.	
Marginal	2.248 (1.346–3.756)	0.002 **			2.226 (1.275–3.886)	0.005 **
Average	2.155 (1.201–3.865)	0.010 *			1.695 (0.893–3.219)	0.107
HPS						
Minimum	ref.		-		-	
Standard	0.784 (0.501–1.228)	0.288	-		-	

COR: Crude Odd Ratio; AOR: Adjusted Odd Ratio; ***: *p* < 0.001; **: *p* < 0.01; *: *p* < 0.05; Grey background: the predictor variable not included in analysis.

**Table 8:** Univariate and multivariate logistic regression of drug abuse with socioeconomic factors in the CHL and FHL models.

Variables	Drugs Abuse					
	Univariate		Model 1 (CHL) Pseudo R^2^ 0.235		Model 2 (FHL) Pseudo R^2^ 0.194	
	COR (CI 95%)	*p*	AOR (CI 95%)	*p*	AOR (CI 95%)	*p*
Gender						
Female	ref.		ref.		ref.	
Male	0.101 (0.022–0.454)	0.003 **	0.084 (0.019–0.384)	0.001 **	0.126 (0.028–0.575)	0.007 **
Grade						
10	ref.		-		-	
11	1.154 (0.349–3.813)	0.814	-		-	
12	0.803 (0.190–3.402)	0.766	-		-	
Academic Performance						
Middle-Low	ref.		-		-	
High	1.688 (0.354–8.057)	0.511	-		-	
Highest	0.629 (0.113–3.498)	0.596	-		-	
Father Education						
No Formal Education	ref.		-		-	
Primary Education	179497207.314 (0.000–0.000)	0.997	-		-	
Secondary Education	7.097 (0.802–62.836)	0.078	-		-	
Higher Education	7.556 (0.799–71.452)	0.078	-		-	
Mother Education						
No Formal Education	ref.		-		-	
Primary Education	0.000 (0.000–0.000)	0.999	-		-	
Secondary Education	0.000 (0.000–0.000)	0.999	-		-	
Higher Education	0.000 (0.000–0.000)	0.999	-		-	
Allowance						
Low	ref.		-		-	
Middle	2.479 (0.615–9.987)	0.202	-		-	
High	0.753 (0.227–2.498)	0.643	-		-	
Internet Access						
No Private Internet	ref.		-		-	
<11 Giga	0.721 (0.191–2.719)	0.629	-		-	
>11G, <Unlimited	25440549.054 (0.000–0.000)	0.995	-		-	
Unlimited	0.841 (0.223–3.167)	0.798	-		-	
CHL						
Inadequate	ref.		ref.			
Problematic	3.587 (0.830–15.504)	0.087	4.636 (0.993–21.634)	0.051		
Sufficient	6.365 (1.543–26.244)	0.010 *	9.304 (2.094–41.327)	0.003 *		
FHL						
Limited	ref.				ref.	
Marginal	14.340 (1.854–110.880)	0.011 *			12.849 (1.654–99.793)	0.015 *
Average	9.358 (1.209–72.459)	0.032 *			6.085 (0.772–47.963)	0.086
HPS						
Minimum	ref.		ref.		-	
Standard	0.419 (0.144–1.217)	0.110	0.304 (0.101–0.915)	0.034 *	-	

COR: Crude Odd Ratio; AOR: Adjusted Odd Ratio; **: *p* < 0.01; *: *p* < 0.05; Grey background: the predictor variable not included in analysis.

## Data Availability

The corresponding author can provide the data used in this study upon request.

## Supplementary Materials

The following are available online at https://www.mdpi.com/article/10.3390/ijerph18158111/s1, Form S1: Indonesian Health Promoting School Program Observation checklist and result.
