# Supplementary material for: Health Literacy and Health Behavior: Associated Factors in Surabaya High School Students, Indonesia
Source: Int J Environ Res Public Health. 2021 Jul 30;18(15):8111. doi: 10.3390/ijerph18158111 (PMC8345632; doi:10.3390/ijerph18158111)
Supplement: Supplementary file 1 [file ijerph-18-08111-s001.zip › ijerph-1275701-supplementary.pdf]

HEALTH PROMOTING SCHOOL OBSERVATION SHEETS
FOR HIGH SCHOOL

A. SCHOOL IDENTITY

1.	School Number	:	....
2.	School Accreditation	:	A / B / C / D *)
3.	School Category	:	Public School/National Private School/International School *)<br>Year ....
4.	The number of students	:	Total .... male ... . female ....
5.	Number of Class	:	....
6.	Number of Classrooms	:	....
7.	Total number of teachers		
	a. Mandatory Subject Teacher	:	....
	b. Physical Education Teacher	:	....
	c. Local Content Teacher	:	....
	d. Religion teacher	:	....
8.	Building Status	:	(Own / Grant / Rent *)
9.	School land area	:	.... m2

**) Choose the appropriate one*

B. IMPLEMENTATION OF HEALTH PROMOTING SCHOOL - HIGH SCHOOL PRIVATE VOCATIONAL SCHOOLS (TRIAS)

1. Health Education

No.	Statement	Yes	No	Info
1.	Physical and Health Education are carried out in a curricular manner			
2.	The teacher makes a health education learning plan			
3.	There is a teacher handbook and reading about health education			
4.	There are health education reading books			
5.	There is a physical education teacher			
6.	Physical and Health Education implemented as extracurricular			
7.	Have health education media (posters and others)			
8.	Have a counseling teacher			
9.	Measurement and recording of physical fitness are carried out			
10.	Implementation of Adolescent Reproductive Health Education, HIV-AIDS and Narcotics on an extracurricular basis			
11.	Physical and Health Education are integrated in other subjects			
12.	Physical fitness tests were carried out			
13.	Have a teacher in charge of Health Promoting School Program			
14.	There is an evaluation of health education			
15.	The active role of "peer educators" / "peer counselors" in health education			
16.	Implemented adolescent reproductive health education, HIV-AIDS and Narcotics in a curricular and extracurricular manner or integrated into subjects			
17.	Have a trained Health Promoting School Program teacher and enough in number			
18.	The existence of a partnership for Health Promoting School program with related agency (Community Health Center, Police, Indonesian Red Cross, Disaster Management Agency, and others Health Related NGO)			

2. Health services

No.	Statement	Yes	No	Information
1.	Carried out teenager health counseling			
2.	Have health screening program			
3.	Measure Height and Weight of student			
4.	Provide with first aid and referral system			
5.	There is supervision school shop/canteen			
6.	Periodical health Examination in every 6 months (including height and weight monitoring)			
7.	Recording the results of health examination and height-weight monitoring at medical record books/card			
8.	Have referral system when needed			
9.	There are trained Youth Health Cadres			
10.	Have counseling services for teenagers			
11.	Regular supervision of school cafeteria stalls			

No.	Statement	Yes	No	Information
12.	Supervision of food vendors around the school			
13.	Have health promoting school funds			
14.	Youth Health Cadres amount already reach $\leq$ 10%			
15.	Adolescent health counseling by "peer educators" / "peer counselors"			
16.	Communication forum activities / focus group discussions of "peer educators" / "peer counselors"			
17.	Number of Youth Health Cadres who have been trained $\geq$ 10%			

3. Healthy School Environment

No.	Statement	Yes	No	Information
1.	Provide water that meets terms health			
2.	Have a place for washing hand			
3.	Have toilets that works			
4.	Provide trash can			
5.	There is a functioning sewerage system			
6.	There is a yard/field			
7.	Have a health promoting school corner			
8.	Have health poster about the danger of cigarettes			
9.	Have health poster about the danger of Drug abuse			
10.	Have drainage system			
11.	Do mosquito prevention program once a week			
12.	Have a canteen / school shop			
13.	Have fence			
14.	Have prayer room			
15.	Existence of supervision for school canteen/shop in a routine manner			
16.	Have secure and sturdy fence			
17.	Have greenery/shade			
18.	Have counseling room			
19.	Have health promoting school room with simple equipment *)			
20.	School Environment free from mosquito flick			
21.	Enforce policy for school area free of cigarettes, drugs and alcohol			
22.	Distance from blackboard to frontline bench 2.5 m			
23.	There are hand washers in several places with running water / faucets and soap			
24.	The facilities for school canteen infrastructure meet health requirements			
25.	There is a clean & healthy canteen officer			
26.	Have thrash bin in each class and rubbish shelter at school			
27.	Have student's and teacher's toilets which fulfill health and cleanliness terms			
28.	Have large enough field for ceremony and exercise			

No.	Statement	Yes	No	Information
29.	There is a safe and beautiful fence			
30.	Have school garden			
31.	Has its own health promoting school room with complete equipment **)			
32.	The creation of schools without cigarettes, free of drugs and alcohol			
33.	Have a healthy, safe and nutritious menu with trained school canteen officer			
34.	Rubbish directly transported and thrown away to the final disposal place out of school			
35.	Toilet ratio for students			
	male: 1: 40			
	female: 1:25			
36.	Have a closed used water discharge system			
37.	There are parks / school gardens that are utilized and labeled (for learning facilities) and processing the results of school gardens			
38.	Classrooms meet health requirements (adequate ventilation and lighting)			
39.	Minimum class density of 1: 1.5-1.75 m2			
40.	Has ideal health promoting school space and equipment ***)			

Note :

UKS room with simple equipment: *)

- Bed
- Weight scales, height gauge, Snellen chart
- First aid kit and simple drugs (betadine, ORS, paracetamol)

UKS room with full equipment **)

- Bed
- Weight scales, height gauge, Snellen chart
- First aid kit and medicines (betadin, ORS, paracetamol)
- Medicine cabinets, reference books, medical record, posters, organizational structure, picket schedule the place washing up hand/sink, student morbidity data.

UKS room with ideal equipment *)**

- Bed
- Weight scales, height gauge, snellen chart
- First aid kit and medicines (betadin, ORS, paracetamol)
- Medicine cabinets, reference books, medical record, posters, organizational structure, picket schedule, hand wash / sink, student morbidity data
- Dental Equipment units
- Examples of body, bone / torso models
- etc.

Surabaya, ... ……………………………… , 2019

School Principal*)

(……………………… ... ………)

NIP / NRK

*). Hand Sign of school principal and school stamp

Table Health-promoting school level in 15 high schools in Surabaya, Indonesia.

	Public School	Private School	Vocational School	Total
Health Education				
Minimum	1 (20.00%)	3 (60.00%)	1 (20.00%)	5 (33.33%)
Standard	2 (40.00%)	0 (0.00%)	1 (20.00%)	3 (20.00%)
Optimum	2 (40.00%)	0 (0.00%)	0 (0.00%)	2 (13.33%)
Perfect	0 (0.00%)	2 (40.00%)	3 (60.00%)	5 (33.33%)
Health Service				
Minimum	5 (100.00%)	5 (100.00%)	3 (60.00%)	13 (86.67%)
Standard	0 (0.00%)	0 (0.00%)	1 (20.00%)	1 (6.67%)
Optimum	0 (0.00%)	0 (0.00%)	1 (20.00%)	1 (6.67%)
School Sanitation				
Minimum	4 (80.00%)	5 (100.00%)	5 (100.00%)	14 (93.33%)
Standard	1 (20.00%)	0 (0.00%)	0 (0.00%)	1 (6.67%)
Health Promotion School (Overall)				
Minimum	5 (100.00%)	3 (60.00%)	2 (40.00%)	10 (66.67%)
Standard	0 (0.00%)	2 (40.00%)	3 (60.00%)	5 (33.33%)
